# Intraoperative MRI without an intraoperative MRI suite: a workflow for glial tumor surgery

**DOI:** 10.1007/s00701-024-06165-0

**Published:** 2024-07-10

**Authors:** Henrik Frisk, Oscar Persson, Michael Fagerlund, Margret Jensdottir, Victor Gabriel El-Hajj, Gustav Burström, Annika Sunesson, Annika Kits, Tomas Majing, Erik Edström, Magnus Kaijser, Adrian Elmi-Terander

**Affiliations:** 1 Department of Clinical Neuroscience, Karolinska Institutet, 171 77 Stockholm, Sweden; 2 Department of Neurosurgery, Karolinska University Hospital, Stockholm, Sweden; 3 Department of Neuroradiology, Karolinska University Hospital, Stockholm, Sweden; 4 Department of Perioperative Medicine and Intensive Care (PMI), Karolinska University Hospital, Stockholm, Sweden; 5 Capio Spine Center Stockholm, Löwenströmska Hospital, Stockholm, Sweden; 6 Department of Medical Sciences, Örebro University, Örebro, Sweden; 7 Institute of Environmental Medicine, Karolinska University Hospital, Stockholm, Sweden; 8 Department of Surgical Sciences, Uppsala University, Uppsala, Sweden

**Keywords:** Intraoperative MRI, Glioma surgery, Workflow

## Abstract

**Background:**

Intraoperative MRI (iMRI) has emerged as a useful tool in glioma surgery to safely improve the extent of resection. However, iMRI requires a dedicated operating room (OR) with an integrated MRI scanner solely for this purpose. Due to physical or economical restraints, this may not be feasible in all centers. The aim of this study was to investigate the feasibility of using a non-dedicated MRI scanner at the radiology department for iMRI and to describe the workflow with special focus on time expenditure and surgical implications.

**Methods:**

In total, 24 patients undergoing glioma surgery were included. When the resection was deemed completed, the wound was temporarily closed, and the patient, under general anesthesia, was transferred to the radiology department for iMRI, which was performed using a dedicated protocol on 1.5 or 3 T scanners. After performing iMRI the patient was returned to the OR for additional tumor resection or final wound closure. All procedural times, timestamps, and adverse events were recorded.

**Result:**

The median time from the decision to initiate iMRI until reopening of the wound after scanning was 68 (52–104) minutes. Residual tumors were found on iMRI in 13 patients (54%). There were no adverse events during the surgeries, transfers, transportations, or iMRI-examinations. There were no wound-related complications or infections in the postoperative period or at follow-up. There were no readmissions within 30 or 90 days due to any complication.

**Conclusion:**

Performing intraoperative MRI using an MRI located outside the OR department was feasible and safe with no adverse events. It did not require more time than previously reported data for dedicated iMRI scanners. This could be a viable alternative in centers without access to a dedicated iMRI suite.

**Supplementary Information:**

The online version contains supplementary material available at 10.1007/s00701-024-06165-0.

## Background

Surgical resection is the primary treatment for gliomas. The surgical outcome impacts the subsequent management of these tumors. Studies have shown that increasing the extent of resection is associated with better outcomes such as longer progression-free survival and overall survival [14, 19, 20]. Incomplete, subtotal resection (STR), may lead to tumor regrowth, while excessive resection may cause neurological deficits.

Intraoperative MRI (iMRI) has emerged as a useful tool in glioma surgery to safely improve the extent of resection. [9] By identifying residual tumor and providing updated imaging for navigation to compensate for brain shift, it may reduce the risk for excessive resection or the need for repeat surgery. [3, 6, 15–17] The use of iMRI is increasing in academic and specialized brain tumor centers. However, iMRI requires a dedicated operating room (OR) with an MRI scanner installed solely for the purpose, dedicated OR and radiology staff, time for imaging and on demand access to efficient image review. [10] Although a dedicated OR may be the best way to perform iMRI, not all hospitals can accommodate such a unit withing their existing OR-buildings. In an international perspective, the costs associated with a dedicated OR are considerable and not necessarily affordable for all centers, especially in low-resource settings.

However, it is possible that the benefits of intraoperative imaging may be achieved, without requiring the installation of MRI scanners in the OR and without the need to reallocate staff from the radiology department. The aim of this study was to investigate the feasibility of using a non-dedicated MRI scanner at the radiology department for iMRI during glioma surgery and to describe the workflow with special focus on time expenditure and surgical implications.

## Methods

### Patient selection and study setting

This was a prospective, non-randomized clinical trial conducted from March 15, 2020, to September 15, 2021. All adult patients (≥ 18 years) who were scheduled for glioma surgery and could undergo MRI, were eligible for inclusion. Patients were identified at the multidisciplinary tumor conference and those who were thought to benefit from iMRI were offered participation in the study. Such cases included low-grade gliomas or gliomas in or adjacent to eloquent areas. After obtaining informed consent, a total of 24 patients were included in the study. Operations were performed during office hours Monday to Friday. Before choosing a date for surgery, MRI availability was cleared with the radiology department. The study hospital is a publicly funded and owned tertiary care center serving a region of roughly 2.3 million inhabitants, and the only neurosurgical center in the region. Medical records and imaging data from digital hospital charts were retrospectively reviewed using the health record software TakeCare (Compu Group Medical Sweden AB, Farsta, Sweden).

The study was approved by the national ethical authority (Dnr 2019–05001).

### Workflow

Before surgery, the hospital’s MRI safety form was filled out and signed by the patient. The form serves to identify any contraindication for MRI. All surgeries were performed in the same 60 m^2^ OR with a mobile operating table (Meera, Maquet AG, Rastatt, Germany), Neuronavigation equipment (Curve, Brainlab, Munich, Germany) and surgical microscope (Kinevo, Zeiss, Germany).

### Anesthesia

Anesthesia was induced using Propofol®, and Rocuronium® was used at a dose of 0,6 mg/kg for muscle relaxation. After intubation and throughout the surgery, total intravenous anesthesia was used with Target Controlled Infusion (TCI) of Propofol® and Remifentanil®. All patients were provided with an arterial line, peripheral intravenous lines, and an MRI-compatible urinary catheter. To prevent coughing during transport to the MRI lab, an additional dose of 20–30 mg Rocuronium® was administered. Patients were manually ventilated during the transport between the OR and the MRI lab. Invasive blood pressure and SpO_2_ monitoring were used during the entire procedure. ECG was not used during transport or in the MRI lab. To facilitate the fast transfer of the patient onto the MRI scanning table, all anesthesiology equipment at the MRI lab were prepared in advance. Long intravenous infusion lines (400 cm) were filled with Propofol® and Remifentanil®, labelled on both ends and pulled through a dedicated tunnel to the MRI lab.

### Surgical procedure

The initial part of the surgery was performed according to the center’s local routine. The patient’s head was fixed in a Mayfield clamp, neuronavigation was used and, after craniotomy, the surgery was performed under the surgical microscope. In some cases, intraoperative neurophysiological monitoring was performed using transcranial and subcortical MEP stimulation.

The procedure for iMRI was initiated when the attending surgeon decided that tumor removal was completed, and the wound would normally be closed. The OR-staff was informed, and the wound closed by temporary sutures of the dura, covering of the craniectomy with Spongostan sheets (Ethicon, Johnson&Johnson, USA), and closing the skin with running 2–0 Ethilon suture before removal of the Mayfield clamp and sterile dressing.

In preparation for patient transfer, all equipment attached to the OR table had been positioned on one side to facilitate patient transfer. All equipment such as cables, monopolar, bipolar, and drill were placed on a sterile table, and all surgical instruments were covered to ensure their sterility for the second phase of the surgery. Then the patient, still under general anesthesia, was transferred from the OR table to a hospital bed. The ultrasound probe and surgical microscope were re-draped and the OR was prepared for the return of the patient.

After performing the iMRI, patients without identifiable tumor remnants were returned to the OR suite and the head was fixed using a vacuum pillow. Intravenous antibiotics were repeated before reopening the wound. After sterile draping the wound was reopened, irrigated and hemostasis was controlled. Thereafter permanent dural closure, fixation of the bone flap, and closure of the soft tissues and skin were performed.

Patients with identifiable tumor remnants on iMRI were returned to the OR suite and the head was fixed in the Mayfield head-clamp using new sterile pins. During the transfer procedure a new neuronavigation plan was created by the attending neurosurgeon after evaluation of the MRI together with two senior consultant neuroradiologists. The patient was then registered in the navigation system using the updated navigation plan. After sterile draping the wound was reopened, and additional tumor resection was performed. All additionally resected regions were sent for separate histopathology analysis. After finalized resection, the surgical wound was closed.

All events and time stamps during the procedure were recorded by a dedicated member of the team and documented in the study protocol. Time stamps were made throughout the surgery, imaging and transportations to allow the subsequent analysis of the time expenditures for all parts of the procedures. Complications during surgery and hospital stay were systematically recorded.

### MRI devices and protocol

The MRI suite is situated three floors down from the operation ward and with a total walking distance from operation room to elevator and from elevator to MRI scanner of approximately 140 m. The iMRI images were acquired on one Signa Premier™ 3 T camera and one Optima™ 450W camera (GE Healthcare, Milwaukee, WI). We used a study-specific MRI protocol containing EPIMix, a sequence encompassing six MR contrasts (T1, T2, DWI, ADC, T2*, and T2 FLAIR weighted images [18], unenhanced Ax T1 3D GRE IR BRAVO (volume of 1 mm thick slices), unenhanced Sag T2 FLAIR 3D CUBE (volume of 1.2 mm thick slices), T2 Ax (4 mm) and enhanced T1 3D GRE IR BRAVO. On occasion, the protocol was extended with a DWI MUSE (4 mm) sequence. Gadolinium contrast was administered before the T2 Ax sequence, after which enhanced T1 3D GRE IR BRAVO and, occasionally, DWI MUSE images were collected. As contrast agent, we used gadoteric acid (Clariscan™, 0.5 mmol/ml, 0,2 ml/kg body weight and with a maximum dose of 20 ml). If no enhancing tumor components were found on preoperative scans, we sometimes abstained from administering Gadolinium.

At days of iMRI examinations, the schedule at the radiology department was planned to perform relatively short scans on at least one of the scanners at the suite during the hours that the iMRI patient could be expected. Since the radiology department was alerted approximately 15–20 min in advance, this allowed for either finishing one of the ongoing examinations, before arrival of the intraoperative patient, or postponing an examination that was about to begin. Thus, the MRI suite was able to perform regular exams while waiting for the iMRI patients and, accordingly, the time the radiology nurse used for the iMRI examination could be held similar to times used for other patients that are scanned under anesthesia such as patients in intensive care [10].

The iMRI images were analyzed at the time of examination on a screen adjacent to the MRI console by two consultant neuroradiologists and the attending neurosurgeon. If resectable tumor remnants were found, a new neuronavigation plan was immediately created by the attending neurosurgeon together with the two neuroradiologists.

For the purpose of the study, tumor components extending into areas deemed inoperable by the neurosurgeon and purposely left untouched, were disregarded in the reporting of residual tumor components.

**Fig. 1:**
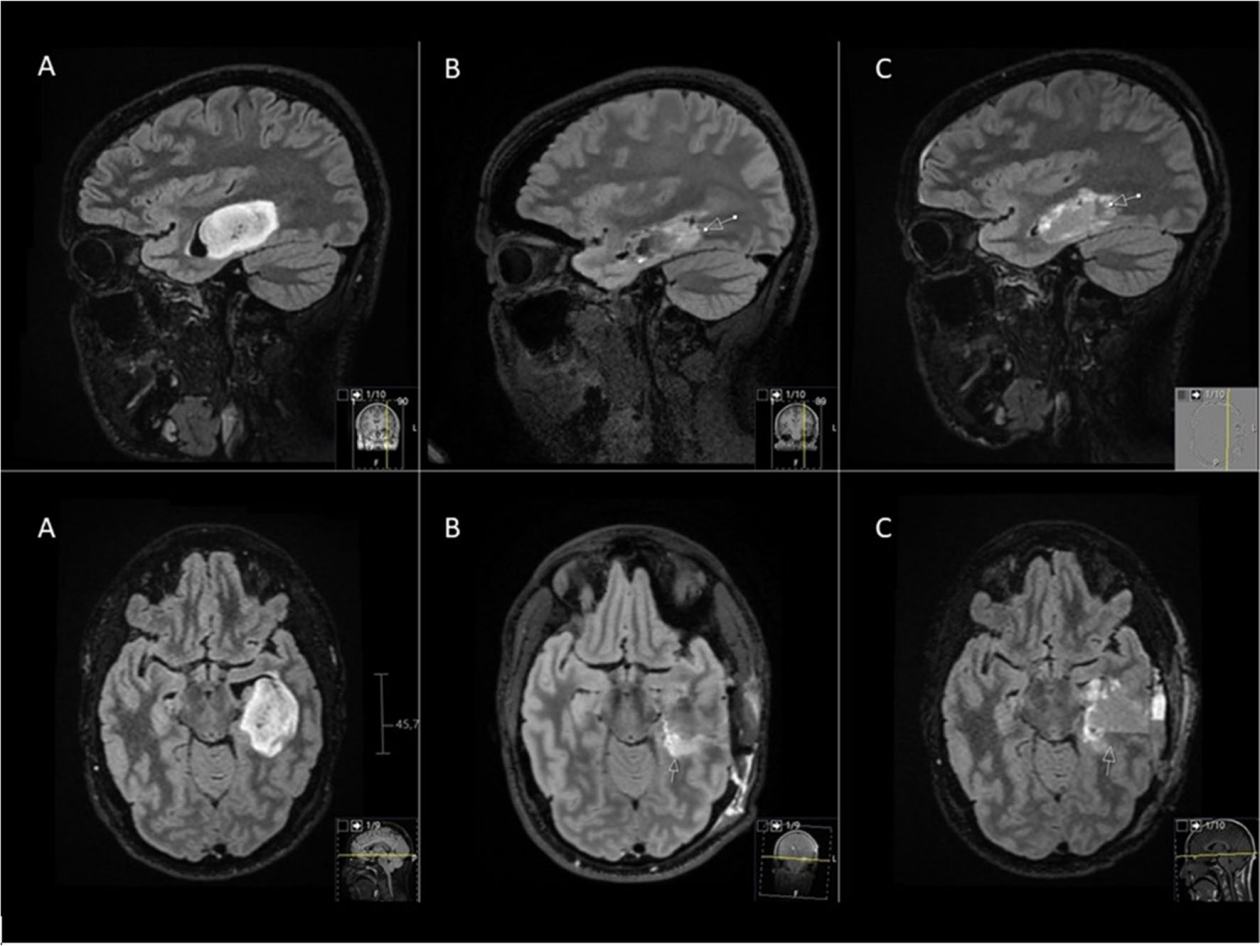
T2 flair 3D Cube images in sagittal and axial projections. A: Preoperative scan showing a non-enhancing mass in the left temporal lobe. B: Intraoperative scan showing a resectable residual tumor in the posterior margin of the surgical cavity (arrows). The patient was taken back to the OR for extended resection. C: Postoperative scan 30 h after surgery showing the residual tumor removed and a fluid level in its place (arrows). Postoperative histological examination showed the tumor to be a grade 2 astrocytoma

All patients, regardless of findings on the intraoperative scan, underwent a postoperative MRI scan within 72 h from surgery on the same scanner that was used for the intraoperative scan. An overall assessment of image quality was performed after acquisition of the postoperative scans (Fig. 1).

### Tumor volumes

Tumors were segmented in the neuronavigation software (Brainlab Elements, Munich, Germany) based on preoperative MRI. For high grade contrast enhancing tumor, outlining was performed on the T1 contrast enhanced volume. For low grade non contrast enhancing tumors, outlining was performed on high signal on T2 FLAIR with manual correction for edema. Potential tumor remnants found using iMRI were outlined on the same MRI sequence as the original tumor volume. All tumor volume calculations were performed using the integrated volumetric tool in the navigation software.

### Time measures and descriptive statistics

All procedural times and timestamps were recorded throughout each procedure by a study team member, solely dedicated to the task. Descriptive data regarding procedural times and tumor volumes are reported as median (range) unless otherwise specified.

## Results

### Procedural times and complications

Twenty-four patients were included in the study: 11 females, 13 males, and the median age was 41.5 (19–64) years (Table 1).

**Table 1:** Included patients and their suspected preoperative diagnoses

	n	%
Sex		
Male	13	54%
Female	11	46%
Age, median 41.5 (19–64)		
20–34	8	33%
35–49	11	46%
50–65	5	21%
Suspected diagnosis		
Low grade glioma	11	46%
Low to high grade glioma	3	13%
High grade glioma	6	25%
Reoperation of known tumor	4	17%

**Fig. 2:**
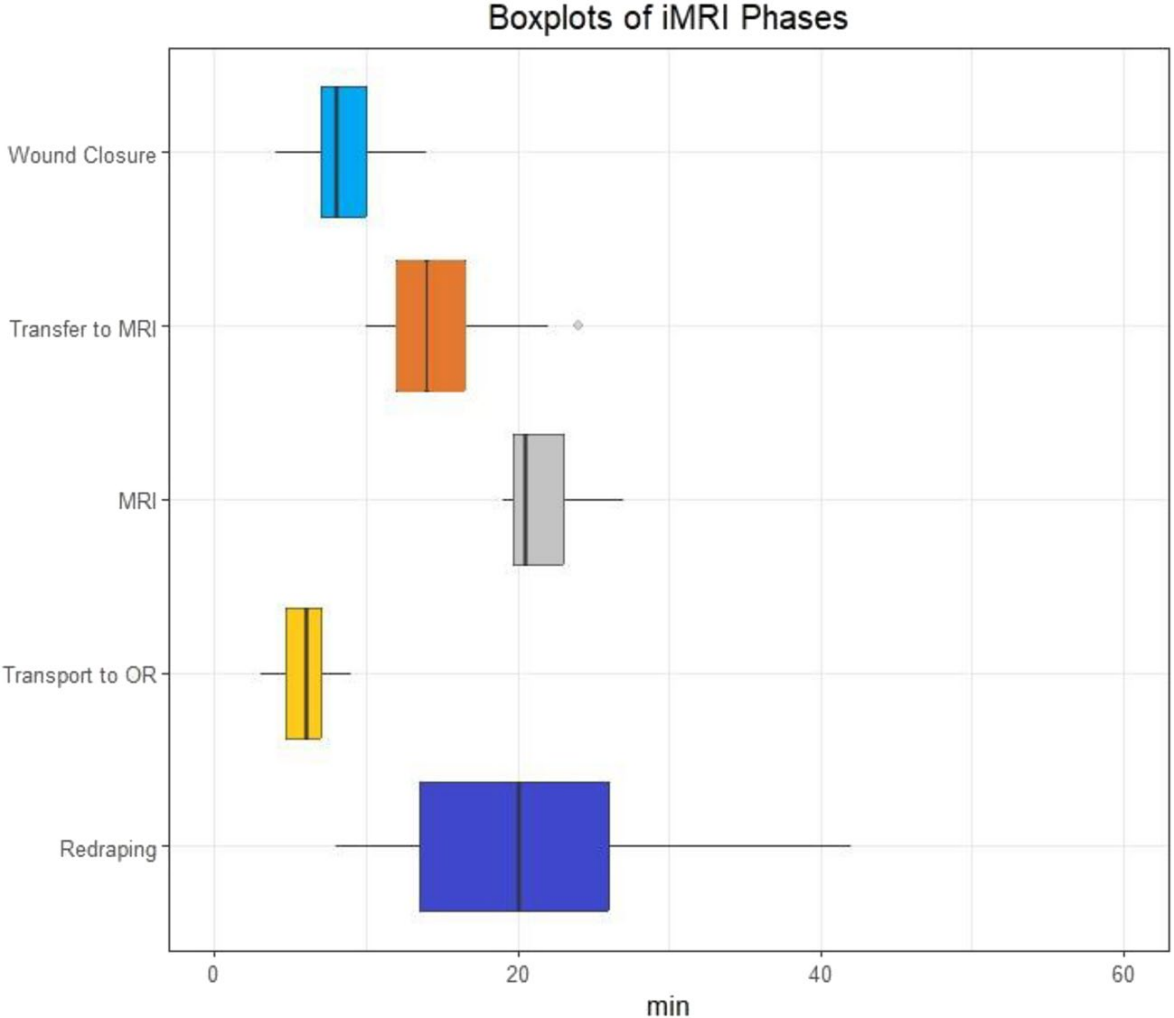
Boxplots illustrating Time expenditure in minutes for each phase of the intraoperative MRI

The median time for the entire iMRI procedure including transfer in the OR, transportation, MRI scan and repositioning in the OR was 60 min (46–98 min). Adding a median of 8 min (4–14) for temporary closure of the wound, the total time from decision to perfrom MRI until reopening of the wound after scanning was median 68 (52–104) minutes. After transfer onto the transportation bed, the median time from departing from the OR suite until re-arrival was 35 (31–50) minutes, with the median transportation time between the OR and MRI lab being 2.5 min (1–6). There was no difference between going to or from the MRI lab. The median time for MRI image acquisition was 20 min (19–27) (Fig. 2).

The median time from return to the OR until reopening was 14 (8–19) minutes for patients without tumor remnants (n = 11), and 26 (17–42) minutes for patients undergoing extended resection (n = 13). Differences in time expenditure for this step were related to the time spent on creating new navigation plans. The median total operation time in all patients (n = 24) including the tumor resection and iMRI procedure was 4:02 h (2:22–6:20).

There were no adverse events during the surgeries, transfers, transportations, or iMRI-examinations. There were no recorded complications during the hospital stay. There were no wound-related complications or infections in the postoperative period or at follow-up. There were no readmissions within 30 or 90 days due to any complication.

### Histopathology and radiology

Analysis of resected tumor material was performed in all cases according to routine protocols at the department of pathology to establish a histopathological diagnosis (Table 2). All intraoperative scans were performed as planned. Of the 24 patients, 13 were scanned on a 3 T scanner and 11 on a 1.5 T scanner.

**Table 2:** Histopathology

Tumor histopathology (n = 24)	n	%
DNET WHO 1	1	4.1
Oligodendroglioma WHO 2	5	20
Astrocytoma WHO 2	8	33
Anapl. Ependymoma WHO 3	1	4.1
Anapl. Oligodendroglioma WHO 3	2	8.3
GBM WHO 4	7	29

As previously mentioned, tumor components extending into areas deemed inoperable by the neurosurgeon and purposely left untouched were disregarded in the reporting of residual tumor components.

Residual tumors were reported on iMRI in 13 patients (54%), in a total of 18 components (2 patients had 2, and 1 patient had 3 residual components). All of them underwent extended resections based on the iMRI findings. The median volume of the components was 3.8 (0.04–9.5) cm3. Histology from the resected areas confirmed the presence of tumor in 9 (69%) of the 13 patients. In the remaining four, the pathology was tumor-negative (gliosis) in two, inconclusive in one, and in one case the separate histopathology from the residual component was missing. Of the two patients where the pathology report did not confirm the intraoperative MRI on the presence of residual tumor, one was performed on the 1.5 T scanner and one on the 3 T scanner. Of the 13 patients that had extended resection, 10 were considered negative for tumor remnants on the postoperative MRI.

In 6 cases parts of the glioma were deemed unresectable and intentionally left untouched. Three of these cases belonged to the group where an extended resection was not performed (negative iMRI group). The other three belonged to the group where an extended resection was performed to remove other, resectable tumor components (positive iMRI group). In the negative iMRI group the first case had a non-contrast enhancing tumor component purposefully left in the insula and thalamus. The second case had a grade II astrocytoma, where a suspected small non-contrast enhancing remnant was left in the splenium of the corpus callosum. The third had a tumor in the premotor area with small non-contrast enhancing remnants adjacent to the subcortical corticospinal tract, which were deemed irresectable based on motor responses in the intraoperative neurophysiological monitoring. In the positive iMRI group, the cases where tumor remnants were intentionally left included one case with a minimal non-contrast enhancing remnant of a low-grade astrocytoma in the genu of the corpus callosum. A second case, operated for a low grade fronto-temporo-insular oligodendroglioma, had small remnants infiltrating into the anterior part of the basal ganglia. A third case, a low-grade astrocytoma in the temporal lobe showed unresectable tumor extension into the trigonum, walls of the occipital horn of the ventricle and crus of fornix.

As expected, residual tumor was detected on the postoperative MRI in all 6 cases. In addition, residual tumor was noted on the postoperative MRI in one more case where the iMRI was negative (Supplementary Table 1).

This patient, with a tumor negative iMRI but a positive postoperative scan, was scanned on a 3 T machine both intra- and postoperatively. On the preoperative scan, part of the tumor was overlooked as it was situated in a gyrus that was compressed against the falx. The deformation of the gyrus remained on the iMRI, but had resolved on the postoperative MRI, where the remnant was relatively easy to recognize on T2 FLAIR.

Assessment of image quality did not suggest any systematic differences between the intraoperative and postoperative scans.

## Discussion

Intraoperative MRI has proven effective for controlling the extent of resection during surgery for primary brain tumors. [23] [5, 8] Although known to significantly extend operative times, the benefits associated with its use have been established to outweigh the risks with extended OR times [7]. Nevertheless, this conclusion is mainly based on findings from studies on mobile iMRI systems or dedicated MRI systems adjacent to the OR, as there are no studies on iMRI using a conventional MRI lab distant to the OR. Our study aimed to determine if performing iMRI using an MRI scanner at the conventional MRI lab located outside the operating department was feasible or more time consuming. Overall, we found that iMRI added a median of 68 min to the total operative time, without any adverse events in terms of complications or infections.

Other studies have found that the use of high field iMRI prolonged the operation time between 72 and 120 min.[4, 12, 13, 21] However, when our results are compared to older studies, it must be acknowledged that we benefit from the development of faster MRI scanning techniques. Relying on a single MRI scanner for iMRI, however, may introduce significant delays in the process. A study by Martin et al., found that problems with the MRI scanner leading to longer operation times occurred in as much as a fourth of the patients [13]. These problems are rarely present when the patient is moved to a radiology ward where several scanners are available. Assessing the true cost in time for performing a temporary wound closure and transporting the patient to and from the MRI suite would require a separate study. However, in this case, the transport of the patient to an MRI lab outside the OR did not prolong the operation time more than what has been reported in studies using a dedicated OR with a mobile or stationary high field MRI system. Additionally, using the conventional MRI lab for iMRI may provide benefits compared to the dedicated intraoperative systems. Arguably, an MRI-lab will be prioritized in terms of investments in the latest technology and to date the available magnetic field strengths of conventional scanners surpass those of iMRI scanners. In addition, mobile MRI systems carry restrictions regarding patient size and morphology, with obese patients or those with short necks and wide shoulders being at a disadvantage [7].

In contrast to traditional MRI scans performed in radiology departments where trained personnel are readily available, utilizing iMRI necessitates specialized personnel or the relocation of radiology staff—a valuable and limited resource in modern hospitals [10]. The significant costs associated with purchasing, installing, and maintaining an iMRI system adjacent to or within the OR may also pose a substantial barrier to acquiring this technology, particularly in resource-constrained settings [1]. Moreover, since individual MRI scanners may be put out of service for a variety of reasons such as routine maintenance, software updates or upgrades, safety concerns, and technical issues, performing intraoperative examinations at the radiology department where several scanners may be available allows for a more secure system for intraoperative MRI exams [13]. In addition, whereas the MRI scanner at the radiology department provides important utility for the entire hospital and perhaps also neighboring health care providers, a dedicated iMRI may not be available for any routine diagnostic examinations. Thus, the performance of iMRI at the radiology department may provide a cost efficient alternative for hospitals that want to offer patients intraoperative MRI imaging without taking on the costs of installing and maintaining an MRI scanner that may be little used. [10]

Compared to performing iMRI at the radiology department, conventional iMRI where the patient remains in the operation room offers several advantages. Most importantly, the patient is not transported outside the operation ward, as patient transportation is recognized to carry risks [11]. However, transportation risks are related to the situation. For intubated patients, the need for anesthesiology and air-way expertise is recognized, as well as good monitoring and compliance to safety checklists [11]. In our study, there were no complications, neither surgical nor transportation related. Although the sample size may have been too small to detect rare complications that may occur outside the operation room, this lack of complications is reassuring and suggests that our protocol may be safely tried by other centers.

Nonetheless, when performing iMRI using an MRI outside the OR department it is crucial to make the transfer process as streamlined as possible to save time. The surgical draping of the patient must be performed in a manner that facilitates easy transfer to the bed, corridors must be kept clear for passage, and elevators made available. Surgical site infections (SSI) are one of the most feared and common complications in neurosurgery. Prolonging the time of a surgical procedure has been shown to increase the risk of developing SSI [2]. Thus, the need for vigilance regarding sterility and aseptic technique cannot be overly emphasized when performing intracranial surgery interrupted by transportation to and from an MRI lab.

Other advantages with conventional iMRI include keeping the head clamp in place, thus not having to redo the navigation registration. Although this step may be time-consuming and tiring for the staff, it poses little risk to the patient. Since the total time needed for iMRI in this study was similar or shorter than conventional iMRI in the literature [4, 12, 13, 21], the study suggests that these disadvantages with MRI outside the operation room can be overcome.

### Quality of iMRI

In this study, we used a dedicated MRI protocol for the intraoperative scans. It is important to notice, however, that the main sequences used for tumor detection, unenhanced and enhanced Ax T1 3D GRE IR BRAVO, unenhanced Sag T2 FLAIR 3D CUBE, and T2 Ax were the same as those used in the routine follow-up of tumor patients. Since the study was designed for other purposes than assessing image quality in scans performed on sedated patients with temporary wound closures versus awake patients with permanent wound closures (a study that would have required a substantially bigger number of patients) no conclusions on image quality regarding intra and postoperative scans can be made based on this study. This applies also for detecting any differences in diagnostic accuracy between intraoperative scans performed on 3 T scanners versus 1.5 T scanners.

The dedicated iMRI protocol was designed to prioritize the most relevant sequences to limit time expenditure. The median scanning time was 20 min. However, if more sequences had been considered necessary, at our disposal were all the sequences in use on GE scanners in the department.

In one of the patients, we found no tumor remnant on the intraoperative scan whereas one was found on the postoperative scan.

The patient was scanned on a 3 T machine both intra- and postoperatively, and the remnant was situated in a gyrus that was compressed against falx by the adjacent tumor on the preoperative scan so that the swelling of the cortex was not noted. The gyrus remained in that position on the intraoperative scan whereas the remnant was relatively easy to recognize on T2 FLAIR when the gyrus was no longer compressed against falx on the postoperative scan.

This cannot be related to the protocol used for iMRI since the same MRI sequence was used both on the postoperative scan that found the tumor remnant and the one that missed it. The reason for missing the tumor is a matter of speculation, but a likely explanation is that the compressed tissue regained its volume gradually during the postoperative phase and because of this was easier to detect on the postoperative scan than on the intraoperative scan. The study was not designed to assess sensitivity and specificity of iMRI performed at the radiology department versus in the operation room. We therefore find the location of the scanner and differences between patient preparation before scanning, an unlikely explanation for why the tumor remnant was missed in this patient.

In two cases, intraoperative examinations detected tumor remnants, yet the subsequent pathology reports revealed only gliosis. Such occurrences are consistent with and reflect the imprecision associated with this modality. In fact, previous studies evaluating the diagnostic accuracy of iMRI as contrasted with pathology reports demonstrated sensitivity values ranging from 41 to 96%, as well as specificity values ranging from 57 to 100% [22].

The cases where tumors were intentionally left behind, illustrate that even with the use of iMRI, gross total resection is not always feasible when the tumor extends into eloquent areas. Discussion of this topic and the extent in which this can benefit the patient would require a larger and more homogenous patient cohort. The chosen end-point in this study has therefore been to report in how many cases the iMRI helped the surgeon to achieve the intended extent of resection, in comparison to how often this was achieved before the iMRI, and how often this failed to be achieved even with the iMRI.

Overall, based on the current study, it can be concluded that from a clinical and radiological perspective, there were no disadvantages with conducting intraoperative MRI scans in the radiology department rather than utilizing a dedicated scanner within the surgical ward. These findings may carry implications on the practice of neurosurgery, especially in resource-limited settings.

### Strengths and limitations

This is a prospective non-randomized study with a limited sample size. Patients were selected to increase the chances of benefiting of the intraoperative examination. However, this bias served to ensure that the study could be performed on a limited number of patients and is not believed to influence the evaluation of feasibility and time-expenditure of iMRI using the conventional MRI-lab. The fact that all surgeries and transportations of patients could be performed without complications may in part reflect a study-effect where all staff is vigilant and careful to adhere to protocols for sterility and safety. This does not take away from the finding however, that iMRI can be safely performed away from the OR.

## Conclusion

Performing intraoperative MRI using an MRI located outside the OR department was feasible and safe with no adverse events. This approach did not add more time to the surgical procedure than previously reported for dedicated iMRI scanners. Using available hospital resources provided a safe and cost-effective alternative to a dedicated MRI adjacent to the OR. The intraoperative image quality did not differ from that of the postoperative scans.

## Comments:

Valuable contribution to the field of neurooncology and demonstrates more than one way to aim for the best surgical result possible in more than one way.

Jane Skjoth-Rasmussen

Copenhagen, Denmark

## Supplementary Information

Below is the link to the electronic supplementary material.

Supplementary file1 (DOCX 73 kb)

Supplementary file2 (DOCX 76 kb)

Supplementary file3 (DOCX 18 kb)

## Data Availability

Data is available upon request from the corresponding author.

## Code availability

Code availability is not applicable.

## References

[1] Arnold TC, Freeman CW, Litt B, Stein JM (2023) Low-field MRI: Clinical promise and challenges, (in eng). J Magn Reson Imaging 57(1):25–44. 10.1002/jmri.28408 36120962 10.1002/jmri.28408 PMC9771987

[2] Cheng H, Chen BP, Soleas IM, Ferko NC, Cameron CG, Hinoul P (2017) Prolonged Operative Duration Increases Risk of Surgical Site Infections: A Systematic Review. Surg Infect (Larchmt) 18(6):722–735. 10.1089/sur.2017.089 28832271 10.1089/sur.2017.089 PMC5685201

[3] Dorward NL, Alberti O, Velani B, Gerritsen FA, Harkness WF, Kitchen ND, Thomas DG (1998) Postimaging brain distortion: magnitude, correlates, and impact on neuronavigation, (in eng). J Neurosurg 88(4):656–662. 10.3171/jns.1998.88.4.0656 9525711 10.3171/jns.1998.88.4.0656

[4] Eid H, Crevier-Sorbo G, Moreau JT, Saint-Martin C, Elzawawi MS, Mousa WA, Dudley RWR, Wilson N (2020) Eight-Year Experience With 3-T Intraoperative MRI Integration in Focal Pediatric Epilepsy Surgery: Impact on Extent of Resection, Residual Volumes, and Seizure Outcomes, (in eng). AJR Am J Roentgenol 214(6):1343–1351. 10.2214/ajr.19.22336 32208007 10.2214/AJR.19.22336

[5] Fountain DM, Bryant A, Barone DG, Waqar M, Hart MG, Bulbeck H, Kernohan A, Watts C, Jenkinson MD (2021) Intraoperative imaging technology to maximise extent of resection for glioma: a network meta-analysis, (in eng). Cochrane Database Syst Rev 1(1):Cd013630. 10.1002/14651858.CD013630.pub2 33428222 10.1002/14651858.CD013630.pub2 PMC8094975

[6] Gerard IJ, Kersten-Oertel M, Petrecca K, Sirhan D, Hall JA, Collins DL (2017) Brain shift in neuronavigation of brain tumors: A review. Med Image Anal 35:403–420 27585837 10.1016/j.media.2016.08.007

[7] Gerlach R, du Mesnil de Rochemont R, Gasser T, Marquardt G, Reusch J, Imoehl L, Seifert V (2008) Feasibility of Polestar N20, an ultra-low-field intraoperative magnetic resonance imaging system in resection control of pituitary macroadenomas: lessons learned from the first 40 cases, (in eng). Neurosurgery 63(2): 272–84; discussion 284–5, 10.1227/01.Neu.0000312362.63693.78 10.1227/01.NEU.0000312362.63693.78 18797357

[8] Golub D, Hyde J, Dogra S, Nicholson J, Kirkwood KA, Gohel P, Loftus S, Schwartz TH (2020) Intraoperative MRI versus 5-ALA in high-grade glioma resection: a network meta-analysis, (in eng). J Neurosurg 134(2):484–498. 10.3171/2019.12.Jns191203 32084631 10.3171/2019.12.JNS191203

[9] Jenkinson MD, Barone DG, Bryant A, Vale L, Bulbeck H, Lawrie TA, Hart MG, Watts C (2018) Intraoperative imaging technology to maximise extent of resection for glioma. Cochrane Database Syst Rev 1:CD012788. 10.1002/14651858.CD012788.pub2 29355914 10.1002/14651858.CD012788.pub2 PMC6491323

[10] Kaijser M, Frisk H, Persson O, Burström G, Suneson A, El-Hajj VG, Fagerlund M, Edström E, Elmi-Terander A (2024) “Two years of neurosurgical intraoperative MRI in Sweden - evaluation of use and costs,” (in eng). Acta Neurochir (Wien) 166(1):80. 10.1007/s00701-024-05978-3 38349473 10.1007/s00701-024-05978-3 PMC10864221

[11] Knight PH, Maheshwari N, Hussain J, Scholl M, Hughes M, Papadimos TJ, Guo WA, Cipolla J, Stawicki SP, Latchana N (2015) Complications during intrahospital transport of critically ill patients: Focus on risk identification and prevention, (in eng). Int J Crit Illn Inj Sci 5(4):256–64. 10.4103/2229-5151.170840 26807395 10.4103/2229-5151.170840 PMC4705572

[12] Kubben PL, Scholtes F, Schijns OE, Ter Laak-Poort MP, Teernstra OP, Kessels AG, van Overbeeke JJ, Martin DH, van Santbrink H (2014) Intraoperative magnetic resonance imaging versus standard neuronavigation for the neurosurgical treatment of glioblastoma: A randomized controlled trial. Surg Neurol Int 5:70. 10.4103/2152-7806.132572 24991473 10.4103/2152-7806.132572 PMC4078446

[13] Martin XP, Vaz G, Fomekong E, Cosnard G, Raftopoulos C (2011) Intra-operative 3.0 T magnetic resonance imaging using a dual-independent room: long-term evaluation of time-cost, problems, and learning-curve effect, (in eng). Acta Neurochir Suppl 109:139–44. 10.1007/978-3-211-99651-5_21 20960333 10.1007/978-3-211-99651-5_21

[14] McGirt MJ, Goldstein IM, Chaichana KL, Tobias ME, Kothbauer KF, Jallo GI (2008) Extent of surgical resection of malignant astrocytomas of the spinal cord: outcome analysis of 35 patients. Neurosurgery 63(1):55–60; discussion 60–1 10.1227/01.NEU.0000335070.37943.09 10.1227/01.NEU.0000335070.37943.09 18728568

[15] Nabavi A, Black PM, Gering DT, Westin CF, Mehta V, Pergolizzi RS, Jr., Ferrant M, Warfield SK, Hata N, Schwartz RB, Wells WM, 3rd, Kikinis R, Jolesz FA (2001) Serial intraoperative magnetic resonance imaging of brain shift, (in eng). Neurosurgery 48(4): 787–97; discussion 797–8, 10.1097/00006123-200104000-00019 10.1097/00006123-200104000-00019 11322439

[16] Nimsky C, Ganslandt O, Cerny S, Hastreiter P, Greiner G, Fahlbusch R (2000) Quantification of, visualization of, and compensation for brain shift using intraoperative magnetic resonance imaging, (in eng), Neurosurgery 47(5): 1070–9; discussion 1079–80, 10.1097/00006123-200011000-00008 10.1097/00006123-200011000-00008 11063099

[17] Reinges MH, Nguyen HH, Krings T, Hütter BO, Rohde V, Gilsbach JM (2004) Course of brain shift during microsurgical resection of supratentorial cerebral lesions: limits of conventional neuronavigation, (in eng), Acta Neurochir (Wien), 146(4): 369–77; discussion 377, 10.1007/s00701-003-0204-1 10.1007/s00701-003-0204-1 15057531

[18] Skare S, Sprenger T, Norbeck O, Rydén H, Blomberg L, Avventi E, Engström M (2018) “A 1-minute full brain MR exam using a multicontrast EPI sequence,” (in eng). Magn Reson Med 79(6):3045–3054. 10.1002/mrm.26974 29090483 10.1002/mrm.26974

[19] Smith JS, Chang EF, Lamborn KR, Chang SM, Prados MD, Cha S, Tihan T, Vandenberg S, McDermott MW, Berger MS (2008) Role of extent of resection in the long-term outcome of low-grade hemispheric gliomas, (in eng). J Clin Oncol 26(8):1338–1345. 10.1200/jco.2007.13.9337 18323558 10.1200/JCO.2007.13.9337

[20] Stummer W, Reulen HJ, Meinel T, Pichlmeier U, Schumacher W, Tonn JC, Rohde V, Oppel F, Turowski B, Woiciechowsky C, Franz K, Pietsch T (2008) Extent of resection and survival in glioblastoma multiforme: identification of and adjustment for bias, (in eng), Neurosurgery 62(3): 564–76; discussion 564–76 10.1227/01.neu.0000317304.31579.17 10.1227/01.neu.0000317304.31579.17 18425006

[21] Sylvester PT, Evans JA, Zipfel GJ, Chole RA, Uppaluri R, Haughey BH, Getz AE, Silverstein J, Rich KM, Kim AH, Dacey RG, Chicoine MR (2015) Combined high-field intraoperative magnetic resonance imaging and endoscopy increase extent of resection and progression-free survival for pituitary adenomas, (in eng). Pituitary 18(1):72–85. 10.1007/s11102-014-0560-2 24599833 10.1007/s11102-014-0560-2 PMC4161669

[22] Van Hese L, De Vleeschouwer S, Theys T, Rex S, Heeren RMA, Cuypers E (2022) The diagnostic accuracy of intraoperative differentiation and delineation techniques in brain tumours, (in eng). Discov Oncol 13(1):123. 10.1007/s12672-022-00585-z 36355227 10.1007/s12672-022-00585-z PMC9649524

[23] Wach J, Banat M, Borger V, Vatter H, Haberl H, Sarikaya-Seiwert S (2021) Intraoperative MRI-guided Resection in Pediatric Brain Tumor Surgery: A Meta-analysis of Extent of Resection and Safety Outcomes, (in eng). J Neurol Surg A Cent Eur Neurosurg 82(1):64–74. 10.1055/s-0040-1714413 32968998 10.1055/s-0040-1714413

