# Supplementary material for: Intraoperative MRI without an intraoperative MRI suite: a workflow for glial tumor surgery
Source: Acta Neurochir (Wien). 2024 Jul 10;166(1):292. doi: 10.1007/s00701-024-06165-0 (PMC11236858; doi:10.1007/s00701-024-06165-0)
Supplement: Supplementary file 1 — Supplementary file1 (DOCX 73 kb) [file 701_2024_6165_MOESM1_ESM.docx]

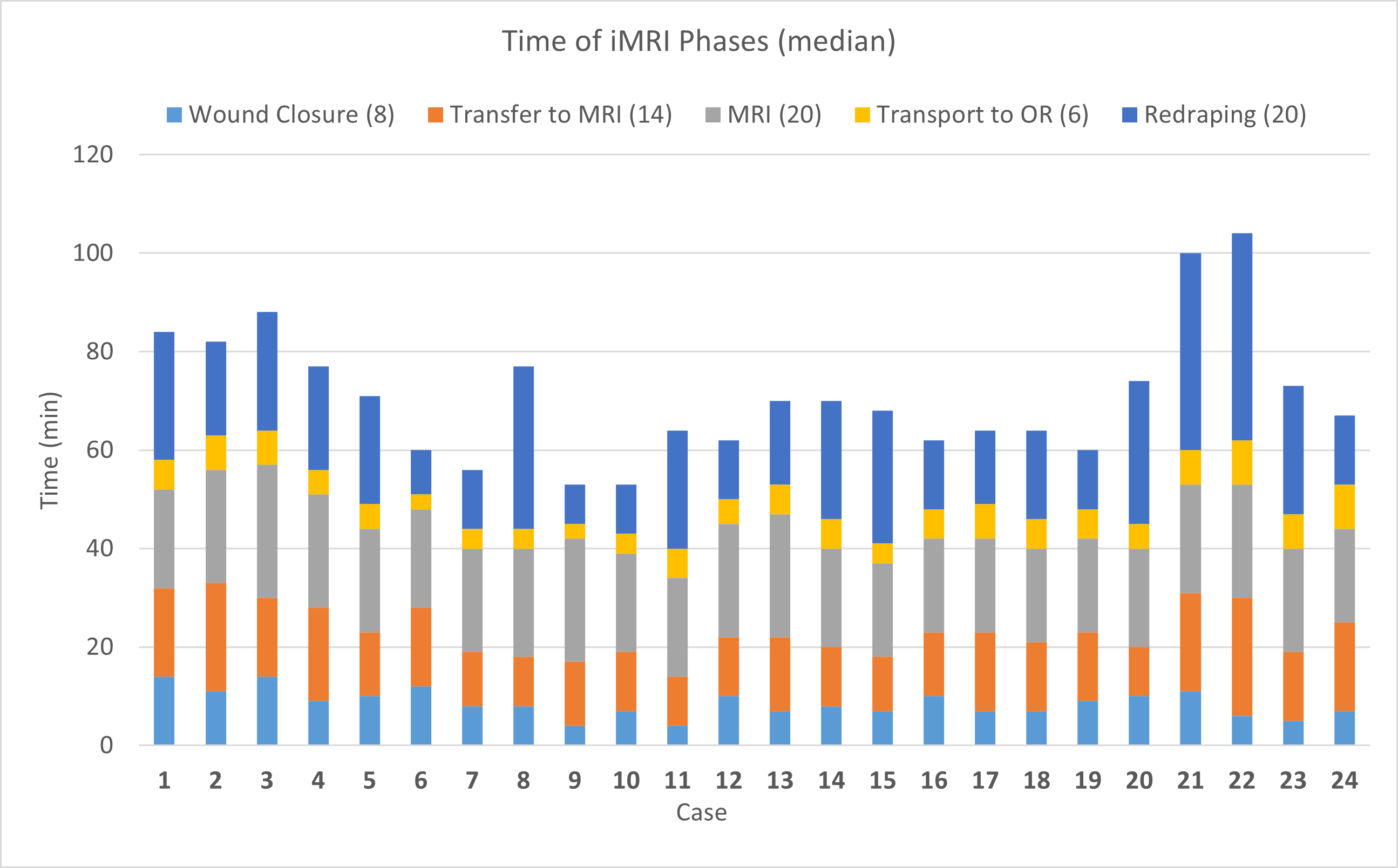


**Supplementary Figure 1.** Individual time expenditure on iMRI phases for each patient in the study
