# Supplementary material for: Intraoperative MRI without an intraoperative MRI suite: a workflow for glial tumor surgery
Source: Acta Neurochir (Wien). 2024 Jul 10;166(1):292. doi: 10.1007/s00701-024-06165-0 (PMC11236858; doi:10.1007/s00701-024-06165-0)
Supplement: Supplementary file 2 — Supplementary file2 (DOCX 76 kb) [file 701_2024_6165_MOESM2_ESM.docx]

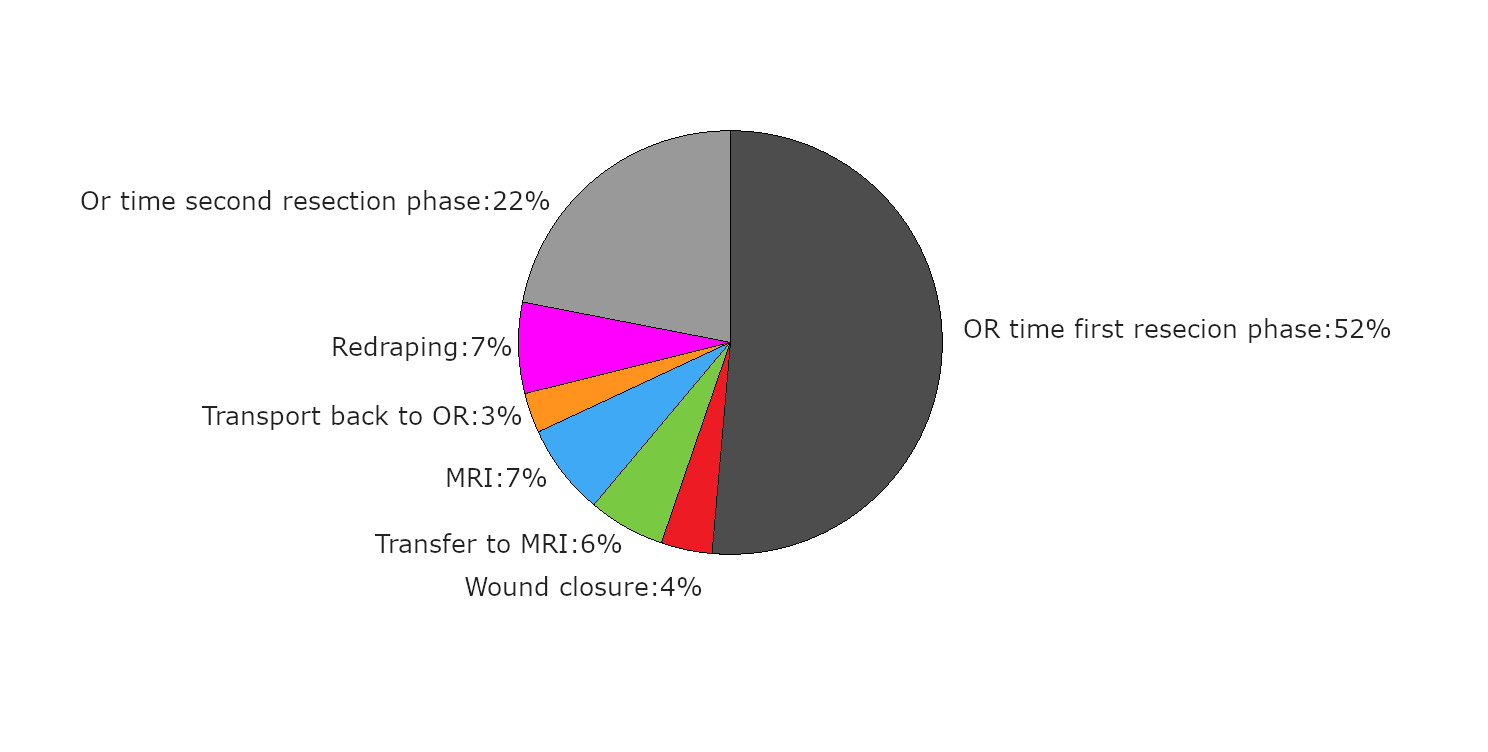


**Supplementary Figure 2.** Median time for all phases of surgery, highlighting the added time by iMRI
