# Supplementary material for: Intraoperative MRI without an intraoperative MRI suite: a workflow for glial tumor surgery
Source: Acta Neurochir (Wien). 2024 Jul 10;166(1):292. doi: 10.1007/s00701-024-06165-0 (PMC11236858; doi:10.1007/s00701-024-06165-0)
Supplement: Supplementary file 3 — Supplementary file3 (DOCX 18 kb) [file 701_2024_6165_MOESM3_ESM.docx]

**Supplementary Table 1.**  Number of positive and negative findings on residual tumor by intraoperative radiology, pathology, and postoperative radiology

| **iMRI findings leading to extended resection** | | |  | **Residual tumor confirmed in pathology report** | | |  | **Residual tumor on postoperative scan** | | |
| --- | --- | --- | --- | --- | --- | --- | --- | --- | --- | --- |
|  | n | % |  |  | n | % |  |  | n | % |
| Yes | 13 | 54% |  | Yes | 9 | 70% |  | Yes | 2 | 22% |
|  |  |  |  |  |  |  |  | No | 7 | 78% |
|  |  |  |  |  |  |  |  |  |  |  |
|  |  |  |  | Gliosis | 2 | 15% |  | Yes | 0 | 0% |
|  |  |  |  |  |  |  |  | No | 2 | 100% |
|  |  |  |  |  |  |  |  |  |  |  |
|  |  |  |  | Inconclusive | 1 | 7.5% |  | Yes | 0 | 0% |
|  |  |  |  |  |  |  |  | No | 1 | 100% |
|  |  |  |  |  |  |  |  |  |  |  |
|  |  |  |  | Missing | 1 | 7.5% |  | Yes | 1 | 100% |
|  |  |  |  |  |  |  |  | No | 0 | 0% |
|  |  |  |  |  |  |  |  |  |  |  |
| No | 11 | 46% |  |  |  |  |  | Yes | 1 | 9% |
|  |  |  |  |  |  |  |  | No | 10 | 91% |
| Total | 24 |  |  |  |  |  |  |  |  |  |
